# A Teleconsultation Device, Consult Station, for Remote Primary Care: Multisite Prospective Cohort Study

**DOI:** 10.2196/33507

**Published:** 2022-05-17

**Authors:** Géraldine Falgarone, Guilhem Bousquet, Arnaud Wilmet, Albert Brizio, Valérie Faure, Celestin Guillouet, Franck Baudino, Isabelle Roque, Samuel Mayol, Frederic Pamoukdjian

**Affiliations:** 1 UMR_S942 MASCOT INSERM Université Sorbonne Paris Nord Bobigny France; 2 Unité de Médecine Ambulatoire Hôpital Avicenne Assistance Publique–Hôpitaux de Paris Bobigny France; 3 Service de Cancérologie Hôpital Avicenne Assistance Publique–Hôpitaux de Paris Bobigny France; 4 H4D (Health for Development) Paris France; 5 Université Paris Cité Paris France; 6 Institut Universitaire de Technologie Université Sorbonne Paris Nord St-Denis France; 7 Service de Médecine Gériatrique Hôpital Avicenne Assistance Publique–Hôpitaux de Paris Bobigny France

**Keywords:** telemedicine booth, primary care, cost-benefit, absenteeism from work, health care system, telemedicine, consultation, remote medical consultation, proof-of-concept, general practice

## Abstract

**Background:**

Telemedicine technology is a growing field, especially in the context of the COVID-19 pandemic. Consult Station (Health for Development) is the first telemedicine device enabling completely remote medical consultations, including the concurrent collection of clinical parameters and videos.

**Objective:**

Our aim was to collect data on the multisite urban and suburban implementation of the Consult Station for primary care and assess its contribution to health care pathways in areas with a low density of medical services.

**Methods:**

In a proof-of-concept multisite prospective cohort study, 2134 consecutive patients had teleconsultations. Consultation characteristics were analyzed from both the patient and practitioner perspective.

**Results:**

In this study, the main users of Consult Station were younger women consulting for low-severity seasonal infections. Interestingly, hypertension, diabetes, and preventive medical consultations were almost absent, while they accounted for almost 50% of consultations with a general practitioner (GP). We showed that for all regions where the Consult Station was implemented, the number of consultations increased as GP density decreased. The study of practitioner characteristics showed GPs from metropolitan areas are motivated to work with this device remotely, with a high level of technology acceptability.

**Conclusions:**

The multisite implementation of Consult Station booths is suitable for primary care and could also address the challenge of “medical deserts.” In addition, further studies should be performed to evaluate the possible contribution of Consult Station booths to limiting work absenteeism.

## Introduction

Alongside the development of the internet and connected tools over the past 2 decades, a rise in the development of eHealth technologies has been observed, facilitating remote communication between patients and caregivers [1,2]. This technological advancement meets the increasing need for more patient-centered medicine. Geographical, temporal, financial, cultural, and digital access issues are at the heart of these changes. Several digital communication systems and devices for telemedicine have been previously reported (eg, interactive voice response, SMS text messages, emails, interactive video, home-based videoconferencing, personal monitoring devices, and personal health records) [1,3-5].

However, telemedicine is not yet ubiquitous and there are ongoing debates on how to improve the quality of patient care. This is particularly true for teleconsultations [6,7]. Some general practitioners (GPs) remain skeptical of telemedicine, with emerging questions on cost-effectiveness, its impact on health outcomes and care, and its usefulness for people with chronic conditions or young, healthy people. For example, in France, the national health insurance has reimbursed teleconsultations since 2019 under specific conditions linked to the standard health care pathway for primary care and GPs [8], but its use was still limited in late 2019. Before the COVID-19 pandemic, many other barriers to adopting teleconsultations worldwide were identified, including barriers related to staff and programmers, patients (age and level of patient education), and practitioners (training, resources, type of device, ethics, confidentiality, and accountability) [6,9].

The year 2020 was seriously impacted by the global spread of COVID-19, which necessitated the promotion of new health care initiatives and a reorganization of telemedicine to meet patients’ expectations for broader access [10-14]. The unstructured and opportunistic implementations of many telemedicine devices and protocols during the pandemic have cast light on the urgent need for standardization [15,16]. In France, the COVID-19 pandemic has accelerated the use of telemedicine, leading to better and wider reimbursement not only for GPs and specialists, but also for nurses, speech therapists, and midwives [17,18].

To date, none of the telemedicine technologies reported involve a single application that enables patients and physicians to conduct a comprehensive measurement of medical parameters. In 2009, Consult Station, a French telemedicine booth, was created and developed by Health for Development (H4D) to meet the growing needs of telehealth; it combines remote consultations, measurement of medical parameters, and diagnostic tools in a single location, and includes a dedicated training program for physicians.

In this proof-of-concept study, we report a multisite implementation of the Consult Station booth for primary care in France and its contribution to health care pathways in the context of generalization of telemedicine devices.

## Methods

### Study Design and Population

This was a multisite prospective observational cohort study that consecutively included all patients aged ≥18 years who had a teleconsultation via Consult Station in France from September 16, 2019, to January 31, 2020, with no exclusion criteria and no patient exclusion in the data analysis.

### Ethics Approval

Informed consent was obtained from each patient before inclusion. Data extraction was anonymized. This noninterventional study obtained the approval of the local ethics committee for collecting and analyzing data (Avicenne hospital, number CLEA-2018-019; 020-019).

### Description of Consult Station

H4D is a company specifically dedicated to clinical telemedicine [19] and it created the Consult Station booth in 2009. This European Class 2 certified autonomous medical device [20] has functions dedicated to the automated measurement of several medical parameters (weight, height, BMI, measures of pain, temperature, blood pressure, cardiac frequency, and oxygen saturation) and includes several diagnostic tools (pain scale, electrocardiogram, stethoscope, dermatoscope, audiometry, capillary glycemia, and otoscope). It has a video interface that enables remote consultations with a physician (Multimedia Appendix 1). There are two modes of teleconsultation: a self-performed checkup and a clinically assisted teleconsultation (deployed in this study). A team of 15 physicians was specifically trained on using the Consult Station booth before the booths were implemented. The physicians’ training program was funded by H4D. Systematic cleansing, adapted to the COVID-19 pandemic, was performed by a trained technical agent between each patient. New booths are to include a UV-C lamp, which shortens the cleansing process to less than 3 minutes.

### Access to Consult Station

Consult station booths were implemented on the premises of large companies and town halls, and employees were informed of the device’s availability and told they had free access to it. When patients wanted a teleconsultation, they had to connect to an appointment booking website provided by H4D and agree to privacy and confidentiality rules. In accordance with the French law on teleconsultations, an appointment must be given to the patient within 48 hours. If necessary, a distant care manager helped the patient schedule the teleconsultation. There were no restrictions on the use of the device and there was no need to be referred by a practitioner to book an appointment.

### GP and Patient Characteristics

GPs were recruited on a voluntary basis and systematically trained. The GP characteristics collected for this study were age, gender, medical specialty, location of private practice, and time devoted to teleconsultations per week.

For each patient, data were collected by the physician during the teleconsultation. Data collected included age, gender, date, location of consultation (ie, Paris, Paris suburbs, or other regions), reasons for consultation, and classified consultation diagnosis according to the International Classification of Diseases, Tenth Revision (ICD-10).

### Statistical Analysis and GP Density Indicators

Categorical data were expressed as numbers and proportions, while continuous data were expressed as mean (SD) or median (IQR) as appropriate.

The number of teleconsultations was assessed according to the local GP density per 100,000 inhabitants [21] and then according to the localized potential accessibility (LPA) to a GP for cities and rural administrative areas [22]. LPA is a composite indicator that considers both GP proximity and GP availability; it is the ratio of the number of completed consultations to the number of available consultations per inhabitant. An LPA value <2.5 per year is used by the French Ministry of Health to define the term “medical deserts” [23].

The data were analyzed and graphics were generated using R statistical software (version 4.0.0; R Foundation for Statistical Computing).

## Results

### Teleconsultation Characteristics

A total of 2134 teleconsultations were carried out from September 16, 2019, to January 31, 2020. The teleconsultations were distributed over weekdays as follows: 419 (20%) on Mondays, 450 (21%) on Tuesdays, 411 (19%) on Wednesdays, 454 (21%) on Thursdays, and 400 (19%) on Fridays. Medical parameters measured and diagnostic tools used were as follows: weight (344/2134, 16%), height (n=344, 16%), BMI (n=344, 16%), temperature (n=1450, 68%), blood pressure (n=1351, 63%), cardiac frequency (n=823, 38.5%), oxygen saturation (n=823, 38.5%), electrocardiogram (n=14, 0.6%), stethoscope (n=896, 42%), dermatoscope (n=156, 7%), and otoscope (n=924, 43%). A teleprescription was issued for 1567 (73%) patients. A sick leave certificate was issued for 42 (3%) patients. Complete data, including the reasons for teleconsultation, were available for 1746 (82%) patients. Overall, 98% (1715/1746) of the teleconsultations were conducted in full, while 2% (n=31) of teleconsultations were abandoned as a result of connection issues. Table 1 shows the distribution of the reasons for teleconsultation. Cough disorders, pain, joint diseases, and rhinitis were the most frequently provided reasons.

**Table 1:** Distribution of the reasons for teleconsultation among 1715 patients.

Reasons for teleconsultation		Patients, n (%)
**Mild infectious diseases**		
	Cough disorders	343 (20)
	Rhinitis	154 (9)
	Fever, unspecified	137 (8)
	Functional urinary symptoms	103 (6)
**Pain**		
	Unspecified pains	187 (11)
	Joint diseases/pain	137 (8)
	Unspecified abdominal pain	51 (3)
	Headache	51 (3)
**Asthenia, skin, and allergy**		
	Asthenia	67 (4)
	Skin disorders	51 (3)
	Unspecified allergy	86 (5)
**Prevention care and certificate**		
	Prescription renewal	51 (3)
	Prevention	120 (7)
	Laboratory results	343 (20)
	Other^a^	154 (9)

^a^Other included unspecified visual disorders (n=19), gynecological disorders (n=17), unspecified vertigo (n=17), pregnancy (n=16), unspecified screening (n=15), nausea or vomiting (n=14), unspecified sleep disorders (n=8), myalgia (n=8), and psychological demands (n=7).

### Use of Consult Station by Women

The main users of Consult Station were younger women with a mean age of 38.7 (SD 10.3; range 20-77) years. Table 2 shows the patient characteristics. The mean teleconsultation duration was 18 (SD 1.2) minutes. Overall, the diagnostic categories most often observed were otorhinolaryngology, osteoarticular pain, and routine clinical examinations, with no difference between women and the whole cohort. Prevention advice (vaccination, laboratory results, and addiction counseling) concerned only 2% (34/1715) of the patients. None of the patients consulted for hypertension- or diabetes-related follow-ups. Referral following a consultation did not occur for 58% (995/1715) of teleconsultations.

**Table 2:** Characteristics of 1715 consecutive patients with teleconsultations.

Variable		Whole cohort (N=1715), n (%)	Women (N=1230), n (%)
**Age cohorts (years)**			
	20-39	948 (56)	722 (59)
	40-59	723 (42)	488 (40)
	≥60	34 (2)	20 (1)
**Gender**			
	Women	1230 (72)	N/A^a^
	Men	475 (28)	N/A
**Diagnostic domains for teleconsultation**			
	Otorhinolaryngology	756 (44)	555 (45)
	Osteoarticular	189 (11)	129 (11)
	Normal clinical examination	187 (11)	111 (9)
	Pneumonology	112 (7)	77 (6)
	Dermatology	77 (5)	66 (6)
	Urology	77 (5)	58 (5)
	Gastroenterology	52 (3)	33 (3)
	Ophthalmology	45 (3)	27 (2)
	Abnormal laboratory results	35 (2)	27 (2)
	Neurology	35 (2)	28 (2)
	Prevention	34 (2)	25 (2)
	Gynecology	29 (2)	24 (2)
	Cardiovascular/high blood pressure	26 (2)	13 (1)
	Psychiatry	18 (1)	15 (1)
	Asthenia	12 (1)	9 (0.7)
	Dental	10 (0.5)	8 (0.6)
	Endocrinology/diabetes	6 (0.3)	4 (0.3)
	Sexually transmitted infection	6 (0.3)	4 (0.3)
	Missing data	8 (0.5)	7 (0.6)
**Consultant recommendations**			
	No orientation	994 (58)	716 (58)
	General practitioner	387 (23)	273 (22)
	Complementary examination	159 (9)	115 (9)
	Specialist	104 (6)	70 (6)
	Other health professional	53 (3)	42 (3)
	Emergency department	10 (0.5)	7 (0.6)
	Missing data	8 (0.5)	7 (0.6)

^a^N/A: not applicable.

### Consult Stations Were Mainly Deployed in Low-to-Moderate GP Density Areas

**Figure 1:**
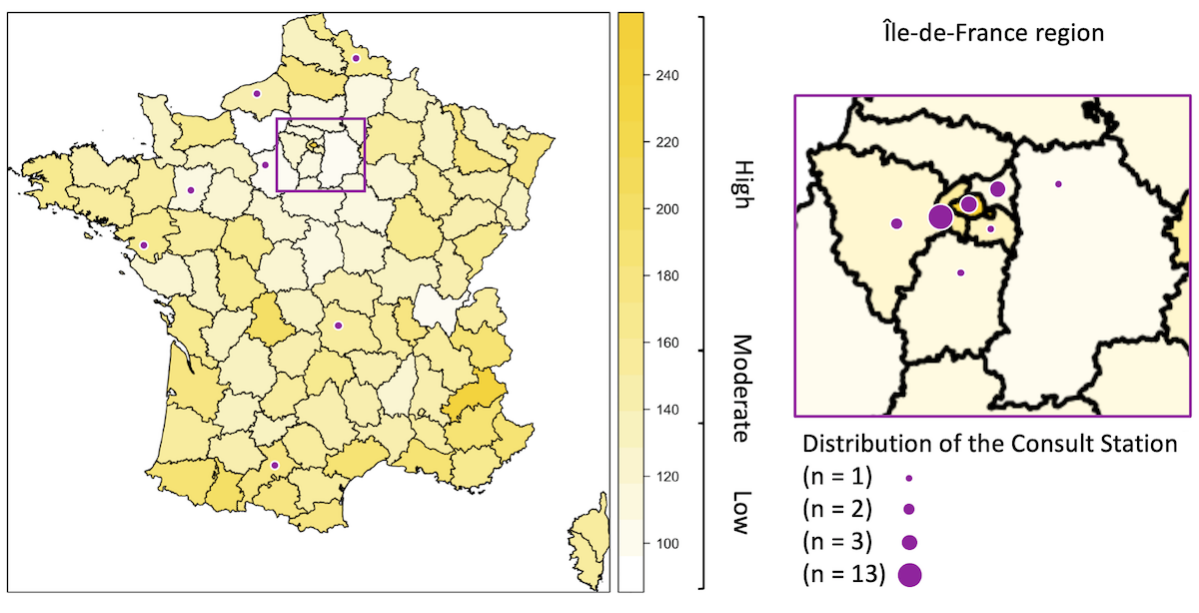
Implementation of Consult Station booths according to general practitioner density in France (left panel) and in the Île-de-France region (ie, Paris and its suburbs; right panel).

A total of 31 Consult Station booths were implemented in France for primary care management, mainly on the premises of large companies (≥5000 employees) and local authorities, with one of them set up inside a town hall (Figure 1). In the Île-de-France region (ie, Paris and its suburbs), 24 (77%) booths were implemented. The GP density of these areas ranged from 96/100,000 to 248/100,000 inhabitants (mean 149.7, SD 27). We classified GP density into 3 categories as follows: low density (96-137), moderate density (138-159), and high density (≥160). We observed that the Consult Station booths were located mainly in moderate-density (16/31, 52%) or low-density areas (11/31, 35%).

We then considered a French composite indicator for access to a GP, namely LPA, which provides the completed number of GP consultations per patient in relation to the number of available GP consultations. Medical deserts are defined by an LPA value under 2.5 per year, which applies to 5.1% of France, while the national LPA value is 3.7 (range 1.4-12.1). Using this threshold of 2.5, none of the Consult Station booths were in a medical desert. We then further classified LPA into 3 categories as follows: low LPA (2.5-3.2), moderate LPA (3.3-4.0), and high LPA (≥4.1). This showed that 19% (6/31) and 55% (17/31) of the Consult Station booths were located in moderate- or low-LPA areas, respectively.

### Consult Station Could Improve Access to Practitioners

Table 3 shows the number of teleconsultations recorded for 28 Consult Station booths according to local GP density and LPA.

**Figure 2:**
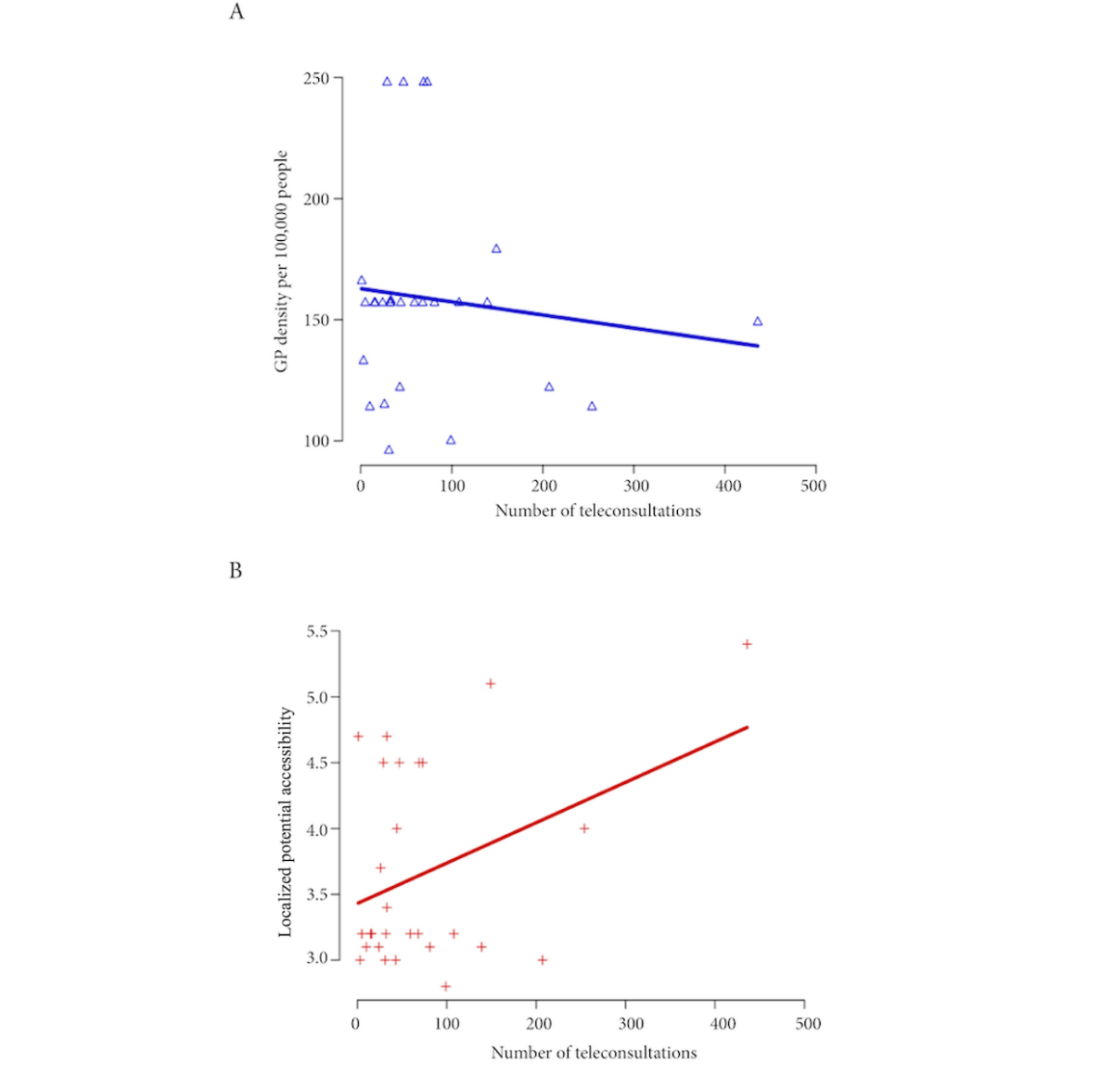
Scatter plot of the number of teleconsultations according to (A) GP density or (B) LPA. GP: general practitioner; LPA: localized potential accessibility.

The number of teleconsultations was high in the Paris suburbs where GP density is low (124 GPs/100,000 inhabitants) and the LPA value is moderate (3.3 consultations/year). Across France, the number of teleconsultations increased as GP density decreased (Figure 2A). In contrast, access to teleconsultation also increased as the LPA indicator increased (Figure 2B). This suggests that access to routine GP consultations was not a hindrance to the use of teleconsultations.

The mean age of the 15 GPs was 39 (SD 8.5, range 30-60) years and 10 (80%) GPs worked in high-LPA areas. The number of years since the GPs’ graduation ranged from 3-35 years. Of the participants, 60% (9/15) worked in a mixed setting, in both private practice and a hospital, and 47% (7/15) worked in a group practice. None had been previously trained for teleconsultations, but 3 of them reported occasional experiences in teleconsultation. Reasons provided by the doctors for their choice to practice telemedicine included the following: the innovative aspect of this device, collaborative work, diversification of their activity, and provision of care to people in medical deserts. For 73% (11/15) of them, the COVID-19 pandemic had not influenced their perception of teleconsultation and 87% (13/15) would recommend teleconsultation to other colleagues. It is worth noting that they were urban practitioners, as none worked in a low-LPA area (Multimedia Appendix 2).

**Table 3:** Number of teleconsultations with the Consult Station according to general practitioner density and LPA (N=2134).

Area	Teleconsultations, n (%)	Mean general practitioner density^a^	C/D ratio^b^	Mean LPA^c^
Paris (center)	222 (10)	High (248)	0.9	High (4.5)
Other regions	660 (31)	Moderate (148)	4.4	High (4.6)
Paris suburbs	1252 (59)	Low (124)	10	Moderate (3.3)

^a^General practitioner density in number per 100,000 inhabitants in France.

^b^C/D ratio: number of consultations/mean general practitioner density per 100,000 people.

^c^LPA: localized potential accessibility.

## Discussion

### Principal Findings

The Consult Station booth is the first telemedicine device enabling completely remote medical teleconsultation with concurrent collection of clinical parameters, as otherwise teleconsultations are often limited to telephone consultations [24-26]. With real-time measurement of several medical parameters, the use of diagnostic tools, and video consultations, Consult Station is a good option when face-to-face consultations are not possible [26]. The COVID-19 pandemic contributed to an acceleration of teleconsultation acceptance and to the restructuring of pre-existing telehealth care devices or pathways. The Consult Station is a particularly original device appropriate for further health care standardization.

In our study, seasonal infections of low severity were the main reason for teleconsultations among younger patients. Interestingly, hypertension, diabetes, and preventive medical teleconsultations were almost absent, whereas they accounted for almost 50% of in-person consultations with a GP in France [27]. The Consult Station could offer a new, convenient health care pathway for younger patients with nonsevere health needs. Further studies are required to determine whether this new, convenient primary care pathway could help reduce visits to hospital emergency departments [28,29]. The results from our study could further the debate on the cost-effectiveness of telemedicine in wealthy countries. Interestingly, only 11% (188/1715) of the patients used the device for a routine clinical examination. Although our proof-of-concept study was not designed to determine whether teleconsultation leaned toward treatment of chronic diseases or more routine conditions, the Consult Station could be of great interest for systematic yearly checkups, particularly in areas with low GP density. Furthermore, a recent study had shown that patients with chronic conditions are open-minded toward alternative modes of telemedicine [11], including their use for treating mental conditions [30].

Most of the patients were younger working women of childbearing age. This gender ratio might be explained by women being overrepresented in the use of the internet and telemedicine [11,15] and because time-saving is a major factor for telemedicine usage [31]. Appointments within 48 hours, convenient health care access comparable to private practice, and flexibility could all contribute considerably to patient satisfaction and acceptance of the Consult Station health care system, as reported with other telemedicine devices [32,33]. Furthermore, the system could help limit absenteeism from work due to illness if booths are implemented in the workplace as in our study [34,35]. In a recent study, the authors found that the rate of absenteeism from work was 3% among 5465 employees, with 56% of absences from women [35]. The rate of sick leave reached 28% overall and was 76% for younger women. In 2018, the annual mean cost of absenteeism from work was estimated at €4059 (US $4460) per individual in France [36], affecting 3.6% of employees. For a large company of at least 5000 employees, this would amount to a cost of €730,000 (US $802,198). In comparison, the minimum annual cost of a Consult Station booth would be €43,320 (US $47,604). This amount includes annual maintenance fees (€10,000, US $10,989), the annual cost equivalent to a full-time technical agent (€21,892, US $24,057) to clean the booth between each patient, and an amortization of the booth over 7 years (€11,428/year, total cost €80,000; US $12,558/year, total cost US $87,912). This could be an advantageous financial operation for companies to prevent work absenteeism. The question of work absenteeism should be addressed in a dedicated study including social and economic patient characteristics.

With the emergence of COVID-19, Consult Station could also be used to help manage patient flows in compliance with barrier measures [37,38].

With a multisite implementation, we believe that Consult Station booths could contribute to addressing the challenge of medical deserts. Even though they were largely implemented on business premises and none were in medical deserts, there was no real bias linked to the geographical distribution of Consult Station booths in our study, since 36% were implemented in areas with low GP density.

From the patients’ perspective, the device offers easy access to doctors even in areas with low GP density. This implies a willingness among practitioners from metropolitan areas to respond to this challenge. Our study results showed a high level of technology acceptability among practitioners and our teleconsultation device addressed several of the barriers previously identified by GPs for the use of telemedicine. With acceptance by both patients and GPs, this type of teleconsultation device provides proof of concept for the generalization of telemedicine, and could succeed where public health policies have failed to address the growing problem of access to care in underpopulated rural areas [36]. Although our study was not designed to evaluate the impact of our device on vulnerable populations, we believe that it does not limit their health care access, as the health care system in France now enables reimbursement for teleconsultations for all patients.

### Conclusions

The multisite implementation of Consult Station booths is suitable for primary care, but it also could meet the challenge of medical deserts. Although various types of telehealth or telemedicine facilities were already available in early 2020, the COVID-19 pandemic has highlighted the need for videoconsultations using remote tools such as those included in the Consult Station. In addition, further studies should be conducted to evaluate the possible contribution of Consult Station booths to limiting work absenteeism.

## Appendix

Multimedia Appendix 1 The Consult Station booth.

Multimedia Appendix 2 Characteristics of the 15 general practitioners who performed teleconsultations.

## Abbreviations

GP: general practitioner
H4D: Health for Development
ICD-10: International Classification of Diseases, Tenth Revision
LPA: localized potential accessibility
