# Correction: A Teleconsultation Device, Consult Station, for Remote Primary Care: Multisite Prospective Cohort Study

**DOI:** 10.2196/43220

**Published:** 2022-10-13

**Authors:** Géraldine Falgarone, Guilhem Bousquet, Arnaud Wilmet, Albert Brizio, Valérie Faure, Celestin Guillouet, Franck Baudino, Isabelle Roque, Samuel Mayol, Frederic Pamoukdjian

**Affiliations:** 1 UMR_S942 MASCOT INSERM Université Sorbonne Paris Nord Bobigny France; 2 Unité de Médecine Ambulatoire Hôpital Avicenne Assistance Publique–Hôpitaux de Paris Bobigny France; 3 Service de Cancérologie Hôpital Avicenne Assistance Publique–Hôpitaux de Paris Bobigny France; 4 H4D (Health for Development) Paris France; 5 Université Paris Cité Paris France; 6 Institut Universitaire de Technologie Université Sorbonne Paris Nord St-Denis France; 7 Service de Médecine Gériatrique Hôpital Avicenne Assistance Publique–Hôpitaux de Paris Bobigny France

In “A Teleconsultation Device, Consult Station, for Remote Primary Care: Multisite Prospective Cohort Study” (J Med Internet Res 2022;24(5):e33507), the authors noted one error.

In the originally published article, author Frederic Pamoukdjian’s name incorrectly appeared as:

Frederic Pamoukjian

It has now been corrected to:

Frederic Pamoukdjian

The correction will appear in the online version of the paper on the JMIR Publications website on October 13, 2022, together with the publication of this correction notice. Because this was made after submission to PubMed, PubMed Central, and other full-text repositories, the corrected article has also been resubmitted to those repositories.

