# Supplementary figures and images for: A Teleconsultation Device, Consult Station, for Remote Primary Care: Multisite Prospective Cohort Study
Source: J Med Internet Res. 2022 May 17;24(5):e33507. doi: 10.2196/33507 (PMC9157322; doi:10.2196/33507)

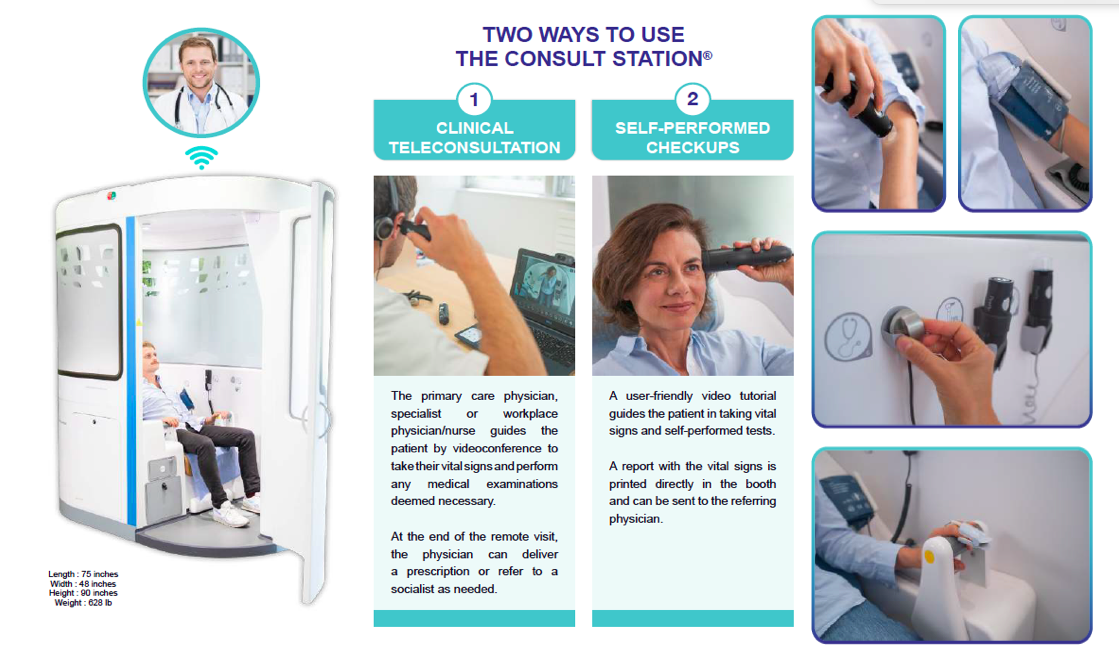

Supplement: Multimedia Appendix 1 [file jmir_v24i5e33507_app1.png]
