# Supplementary material for: A Teleconsultation Device, Consult Station, for Remote Primary Care: Multisite Prospective Cohort Study
Source: J Med Internet Res. 2022 May 17;24(5):e33507. doi: 10.2196/33507 (PMC9157322; doi:10.2196/33507)
Supplement: Multimedia Appendix 2 [file jmir_v24i5e33507_app2.docx]

**Multimedia Appendix 2.** Characteristics of the 15 general practitioners who performed teleconsultations.

| Variables | | Median (IQR, range) | Number of general practitioners, n (%) |
| --- | --- | --- | --- |
| **Age cohorts (years)** | | | |
|  | 30-39 | N/A^a^ | 10 (67) |
|  | 40-49 | N/A | 3 (20) |
|  | ≥50 | N/A | 2 (13) |
| **Gender** | | | |
|  | Men | N/A | 10 (67) |
|  | Women | N/A | 5 (33) |
| **Specialty** | | | |
|  | General practitioner | N/A | 10 (67) |
|  | Emergency doctor | N/A | 5 (33) |
| **Practice in relation to LPA^b^ areas** | | | |
|  | Medical desert (<2.5) | N/A | 0 |
|  | Low LPA (2.5-3.2) | N/A | 0 |
|  | Moderate LPA (3.3-4.0) | N/A | 3 (20) |
|  | High LPA (≥4.1) | N/A | 12 (80) |
| Teleconsultation hours per week | | 6.0 (3.0-7.7, 3.0-9.0) | N/A |

^a^N/A: not applicable.

^b^LPA: localized potential availability.
